# A decade of CD4+ chimeric antigen receptor T-cell evolution in two chronic lymphocytic leukemia patients: were chronic lymphocytic leukemia cells present?

**DOI:** 10.37349/etat.2023.00186

**Published:** 2023-10-31

**Authors:** Dimitrios Bouzianas, Stella Bouziana

**Affiliations:** Kumamoto University, Japan; ^1^Independent Researcher, BReMeL, Biopharmaceutical and Regenerative Medicine Laboratories, PC555 34 Thessaloniki, Greece; ^2^Department of Hematology, King’s College Hospital, SE59RS London, UK

**Keywords:** Chimeric antigen receptor T-cells, chronic lymphocytic leukemia, immunotherapy, long-term remission, CD4+/CD8+ T-lymphocytes

## Abstract

On Feb 2, 2022, *Nature* published the paper titled “Decade-long leukemia remissions with the persistence of CD4+ CAR T-cells” (*Nature*. 2022;602:503–9. doi: 10.1038/s41586-021-04390-6). According to the results presented, it could be argued that “chimeric antigen receptor (CAR) T-cells can actually cure patients with chronic lymphocytic leukemia (CLL)”. CAR T-cells remained detectable more than ten years after infusion, and immunoglobulin heavy chain (IGH) rearrangement deep sequencing showed persistent deep molecular remission for both patients (no CLL clonotypes were detectable six months after CAR T-cell infusion and onwards). However, the existing actual disease status of both patients remained unclear, as it was unknown: (1) if CAR T-cells killed all leukemia cells during the initial anti-leukemic response phase, that is, soon after CAR T-cell infusion into both patients; (2) if few CLL cells survived, but persistent CAR T-cells had been able to destroy any leukemia cells before they reach detectable levels. In the first case, both patients could be considered definitely cured; in the second not and their decade-prolonged deep remission could be a consequence of the cytotoxic activity of the functionally active CD4+ CAR T-cells. The first version appears to be stronger and the supporting arguments have been included in a comprehensive commentary article. A new therapeutic intervention may emerge with the potential to fully improve the quality of life of both patients and in addition, ongoing research into CAR T-cells may turn in a new, more effective direction.

## Introduction

Immunotherapy has become a standard treatment for many cancers, with chimeric antigen receptor (CAR) T-cell therapy representing one of the most promising options. Since 2017, six CAR T-cell commercial products have been approved by the Food and Drug Administration (FDA) to treat highly refractory hematological malignancies. This therapy has been extraordinarily successful putting hematologic patients who had months to live, into remission [1, 2]. However, although thousands of patients have received CAR T-cells, prolonged complete disease remissions are rarely achieved and how long CAR T-cells continue to be functional remains elusive [3]. CAR T-cell therapy frequently remains a last resort to be used when all other treatments have failed, because it’s accompanied by several challenges such as laborious manufacturing process, high costs, and serious toxicities [4].

Chronic lymphocytic leukemia (CLL) is the most common adult leukemia, largely incurable. Durable CAR T-cell activity in CLL is limited and identifying ways to achieve long-term remissions is critical to CAR T-cell utility [5]. The first reports mentioned that only about 25–35% of CAR T-cell recipients with CLL experienced complete remission (CR) [6]. Currently, clinical expansion and functional persistence in CLL are considered key characteristics to ensure long-lasting responses [7, 8]. However, so far little is known regarding the duration and biological behavior of persisting CAR T-cells in patients with prolonged disease remission. Recently, Melenhorst et al. [7] presented a CAR T-cell characterization using sophisticated technologies and tried to identify CAR T-cell properties associated with a decade of remission and anti-leukemic response. In this commentary article, arguments are provided for the potential cure of both patients within the first six months after CAR T-cell infusion.

## Decade-long persistence of active CD4+ CAR T-cells in two CLL patients

Melenhorst et al. [7] presented results of anti-CD19+ CAR T-cells at different time points, which were infused in two CLL patients back in 2010. They brought to light the surprising finding of descendent cells of the initially infused CAR T-cells to circulate in the patient’s blood, accompanied by minimal residual disease (MRD) negative CR. CR was achieved within months and cells patrolled the blood and lymphoid tissues for a decade; in addition, leukemia signs and symptoms disappeared for more than a decade, which correlated with sustained severe B-cell aplasia. It is the first time that such a long-term “remission” has been observed and researchers declared that both patients were deemed cured [7, 9].

## Two response phases: kinetics of CAR T-cell expansion and persistence—temporal analysis of cell composition

**Figure 1:**
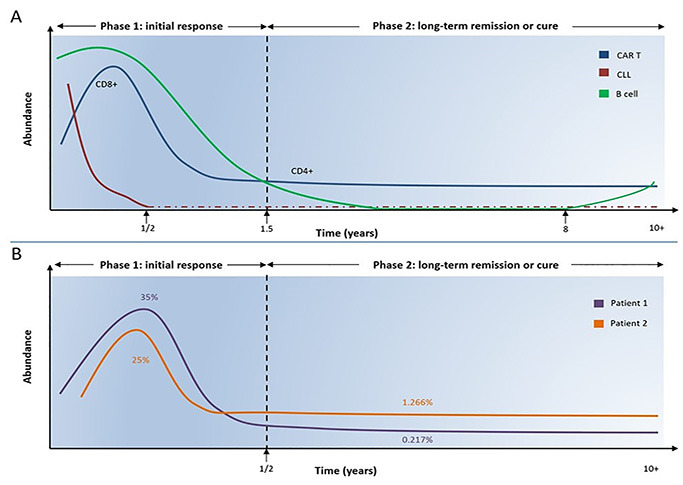
Temporal analysis of cell composition in two CLL patients. A. Cytotoxic CD8+ CAR T-cells expanded and dominated in the initial response phase mediating the killing of CLL cells, which remained undetected six months after CAR T-cell infusion. CD8+ CAR T-cells gradually declined and shifted to CD4+ CAR T-cells, which dominated at low levels over the second phase. In parallel, normal CD19+ B-cells gradually declined resulting in B-cell aplasia, while in the first patient B-cells gradually restored at 8 years post CAR T-cell infusion without disease recurrence. The actual CLL cell status remained unclear after six months, namely, whether the CLL burden was completely eradicated during the initial phase or some residual CLL cells remained over a decade and were destroyed by very few patrol-activated CD4+ CAR T-cells; B. temporal analysis of cell composition in two CLL patients. Estimation of the mean percentage of persisting CAR T-cells out of overall T-cells over two distinct phases in two CLL patients (based on Figure 1b, c [7] and Figure 2a [1], Figure 1b, c is a figure of [7], Figure 2a is a figure of [1], not this article). During the first six months post CAR T-cell infusion, the mean percentage of CAR T-cells out of all T-cells was approximately 35% in patient 1 and approximately 25% in patient 2. The corresponding percentages in the second phase were 0.217% in patient 1 and 1.266% in patient 2

The mapped clonal CAR T-cell evolution over 10 years revealed two distinct response phases [5, 7, 10]: a 1.5-year duration initial acute phase dominated by cytotoxic CD8+ CAR T-cells, which expanded and subsequently contracted in line with reduced CD19+ B-cell numbers. The ensuing long-term remission phase was dominated by low levels of activated CD4+ CAR T-cells and highly suppressed CD19+ B-cells, much less than 1% of peripheral cells. The immunoglobulin heavy chain (IGH) analysis confirmed the leukemic clone was undetectable six months post-infusion (extended data Table 1 of [7], Figure 1A).

**Figure 2:**
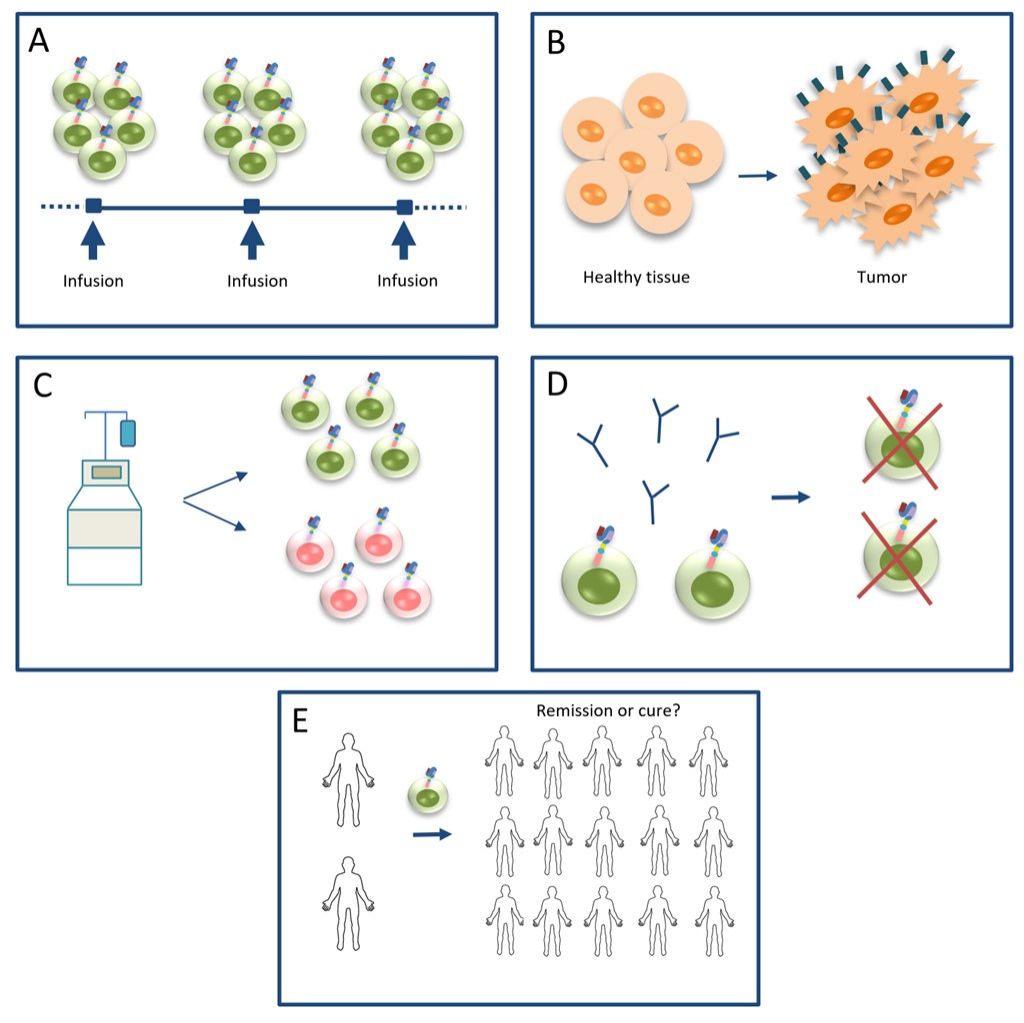
Recommendations for future research and clinical applications in the CAR T-cell field. A. CAR T-cells should be administered in high doses and in a split-dosing manner for safety reasons; B. research should shift to detect tumor-specific antigens and invent more sensitive techniques to facilitate the interpretation of results; C. the CAR T-cell starting material should be defined in terms of composition of CAR T-cell subpopulations; D. anti-idiotypic antibodies should be developed and administered in potentially “cured” CLL patients who are under high risk of infections, to eradicate remaining CAR T-cells and restore B-cell aplasia; E. remission or cure? larger cohorts are needed to confirm the clinical status across other CLL patients, as well as other malignancies

During this phase, CAR T-cell population shifted gradually over time from CD8+ to CD4+ T-cells. Based on Figure 1b, c [7] and Figure 2a [1] (Figure 1b, c is a figure of [7], Figure 2a is a figure of [1], not this article) we estimated the mean percentage of persisting CAR T-cells out of overall T-cells over time-points. We divided the kinetic curve of each patient into two distinct time periods having the 6-month time point post-infusion as a reference point: the first period from infusion until 6 months and the second from 6 months and onwards. In the patient 1, the mean percentage of CAR T-cells was approximately 35% and 0.217% out of all T-cells during the first and second time periods respectively; in the patient 2 the corresponding frequencies were approximately 25% and 1.266% (Figure 1B).

In both patients, the long-term remission phase was dominated exclusively by CD4+ Ki67 long-lived memory CAR T-cells, presenting activation and memory markers. Cells remained metabolically active, expressed tumor-killing molecules (granzymes-perforin), and retained high proliferation capacity [5, 7]. Although they exhibited exhaustion-related inhibitory markers, they remained functionally active rather than exhausted for a decade [7, 8]. The first patient’s CAR T-cells presented cytotoxic activity when confronted with leukemia cells in *in vitro* conditions. IGH deep sequences supported the concept that B-cells continued to be produced by the bone marrow and were exported to the peripheral blood. CD19 expression, notably absent on the stem cells and B-cell precursors, emerges around the late pre-pro-B to pro-B cell stage to mature B-cells; therefore, it seems unlikely the ongoing B-cell suppression was due to eradication of an early progenitor population [11]. The sustained activation of Ki67^hi^ cells was most likely driven by cognate CD19 antigenic signaling of nascent B-cells or B-cell progenitors, that progressed beyond the pre-pro-B-cell stage; B-cells were rapidly killed in an ongoing fashion by the activated CAR T-cells, resulting in long-lasting severe B-aplasia in both patients [8].

## Discussion

Despite the technically intriguing report on the persistent CAR T-cells nature, the actual disease status of patients remained unclear. Did CAR T-cells destroy all CLL cells during the initial phase or did a few survive, proliferate and subsequently be killed by persistent CD4+ CAR T-cells before reaching detectable levels? In the first case, patients could be considered cured (long-term remission couldn’t be attributed to intrinsic CD4+ CAR T-cell cytotoxicity); in the second case, the decade-prolonged deep remission could be a consequence of CD4+ CAR T-cell cytotoxicity (theoretical risk of disease relapse).

The decade-persisting effector CD4+ CAR T-cells raised the suspicion that they were primarily responsible for the decade-deep “remission” [7]. The gradual shift likely arose as a natural functional evolution of both infused CAR T-subpopulations and the temporal predominance of CD4+ cells could be attributed to the following [3, 12, 13]: (1) the total number of peripheral CD8+ T-cells is significantly lower than CD4+; (2) both naive CD4+/CD8+ T-cells decrease in absolute numbers with increasing age, the latter more drastically (patients were 65 years old and 64 years old); (3) preclinical and clinical models have demonstrated that prolonged response by CD4+ CAR T-cells can be attributed to comparable higher exhaustion of CD8+ T-cells.

During the first 6 months approximately 25–35% of functional CAR T-cells out of all T-cells were necessary to induce a deep remission in both patients. In the second period, a much lower percentage of 0.217–1.266% could sustain a durable CR for a decade. This huge difference raises the question “How unique these very few CD4+ cytotoxic patrol cells could be as part of patients’ immune system so that, residual CLL cells have never escaped from their surveillance, even for a whole decade?”. This is implied by the author’s declaration “CD19+ B-lymphocytes and CLL cells had remained undetectable or highly suppressed (less than 1% of cells) beyond 3 years post-infusion” [7]. In line with the prevailing notion that long-term remission necessitates persisting cytotoxic CAR T-cells, the authors sought to identify the characteristics of long-term CD4+ cells with a view of potential further application as prognostic markers. However, while CD8+ CAR T-cells played a critical role in early disease elimination, it remained unknown if cytotoxic CD4+ CAR T-cells were implicated in long-term remission. Dr. Carl June, the principal investigator of this trial, made the following remarkable statement to the New York Times on Feb 02, 2022, which reflects the authors dilemma regarding the CLL prolonged presence: “We cannot find any leukemia cells, perhaps they are still in tiny quantities and emerging only to be knocked back by cytotoxic CD4+ CAR T-cells, like whack-a-mole, or they might have been finally eradicated back in 2010” [14].

Another open question of debate relates to how long CAR T-cells should persist in order to achieve a “cure” in an otherwise “incurable” cancer [8]. Dr. Carl June estimates that since 2010 tens of thousands of patients have received CAR T-cell therapy [4], with an estimated 4 in 10 going into remission, but the duration of remission and CAR T-cell persistence remains elusive. Melenhorst et al. [7] showed that the progeny of infused CAR T-cells circulated in the patients’ blood for a decade and the increase in B-cells at 8 years (Figure 1b [7], and Figure 1b is a figure of [7]) could be attributed to a decrease in the cytotoxic potential of functional CD4+ CAR T-cells (exhaustion or onset of senescence). Remarkably, genetically modified T-effector (TEFF) cells, TEFF memory (TEM), T-central memory (TCM), and T-stem cell memory (TSCM) have been detected in patients’ blood up to 12–14 years after infusion [15, 16]. Around this time B-cells may fully recover due to the possible disappearance of CD4+ CAR T-cells and such an outcome would represent a strong confirmation of our “cure” speculation.

If CLL cells were finally eradicated back in 2010 [14], in the long phase the CD4+ cells destroyed only the natural B-cells and it could be argued that their “anti-leukemic properties” were studied with no leukemia cells. Based on Figure 1b [7] (Figure 1b is a figure of [7]), B-cells recovered in patient 1 between 8–10 years and earlier between 2–4 years with MRD negativity (Figure 1b [7], and Figure 1b is a figure of [7]). Could the recovered cell pool at both time-points contain CD19+ leukemic cells (≤ 1 × 10^‒6^)? If so, what prevented them from causing disease recurrence as it usually happens? Why in thousands of similar cases the usual outcome is relapse after a period of remission, at best after a few years [4]? It seems a logical approach to accept that most likely all CLL cells were completely eradicated during the first 6 months and patients were entirely cured. This view could be reinforced by the aforementioned data on the proportion of persistent CAR T-cells over the two distinct time-periods. However, given that the authors did not evaluate other tissues apart from blood, there is a theoretical possibility that a few CLL cells remained in lymphoid tissues after the first 6 months which were then killed by the CAR T-cells but in the absence of evidence, reliable scientific comments cannot be made. However, accepting this scenario the same question arises “Why the disease did not recur later?”, reinforcing the possibility of early disease eradication. Under this assumption, we recommend the following for future CAR T-cell research and clinical applications (Figure 2).

Cure or decade-long CR? Summary of arguments in favor of the first version:

(1) Extremely large difference in the proportion of functional CAR T-cells between the two distinct time-periods: 25–35% *vs.* 0.217–1.266% to sustain a durable deep CR for 6 months and a decade, respectively.
(2) Surprising anti-leukemic capacity of very few persistent CD4+ CAR T-cells to prevent disease recurrence, by highly suppressing residual CLL cells for a whole decade.
(3) Tens of thousands of patients have been treated with CAR T-cells since 2010, however, relapse after months is the usual outcome (at best, only in a minority of cases does remission last a few years).
(4) Natural expected temporal shift of CD8+ to CD4+ CAR T-cells.
(5) Increased proportion of normal B-cells at approximately 8 years after infusion could be attributed to CAR T-cell exhaustion or senescence. Potential B-aplasia and CD4+ CAR T-cell disappearance around 14 years without disease recurrence could confirm the cure hypothesis/speculation.

### Administration of high doses and in a split-dosing manner

A similar clinical (time-frame) with both patients was reported in a young acute lymphoblastic leukemia patient, whose malignant cells became undetectable when treated with the same CAR construct and a very high infused dose [2]. In all three cases, possibly the leukemia was eradicated by chance because of the very high doses. The targeted cure is not favored by the current strategy, as CD19 is not exclusively expressed on B-leukemic cells. There is no scientific criterion of calculating an individualized therapeutic dose, and very high doses of CAR T-cells may cure only randomly and applying a split-dosing regimen for safety reasons.

### Research should shift to detecting tumor-specific antigens

CAR T-cells are incredibly potent in killing tumor cells and both academics and biotech research focus on improving manufacturing methods to make them more potent (increasing *in vivo* expansion and persistence) and less toxic [8, 9]. Instead, to facilitate the interpretation of results research should be directed towards the detection of antigens exclusively expressed only on malignant cells and not on normal cells, and the invention of ultra-sensitive techniques, e.g., next generation sequencing (NGS) detecting tumor cell concentrations ≤ 1 × 10^‒7^ or ≤ 1 × 10^‒8^, etc.

### Defined composition of CAR T-cell subpopulations

CAR T-cell products contain a mixture of CD4+, CD8+, and nonconventional CAR T-cells. As CD8+ T-cells are less durable, a collection with fewer CD4+ TNAIVE/TSCM cells could show equivalent (or higher) cytotoxicity to a similar CD8+ collection containing equal or more cells. The phenotype and amount of infused cells may influence the long-term persistence of adoptively transferred cells [3, 17]. Human TSCM cells have a privileged role in T-cell persistence, as they positively correlate with early expansion and absolute counts of dominant long-term T-cell clonotypes [15, 16]. Future studies should quantify the amount of individual CAR T-cell subsets after *ex vivo* expansion to better explain results, e.g., the role of CD8+/CD4+ CAR T-cells to control MRD at different time points.

### Anti-idiotypic antibodies to eradicate B-cell aplasia in potentially “cured” CLL patients

The prolonged risk of infections and severe dependence on immunoglobulin monthly administration reduced patients’ quality of life [one patient succumbed to coronavirus disease 2019 (COVID-19) infection in 2021] [18]. Anti-idiotypic antibodies [19] would be an ideal “antidote” to restore B-cell aplasia depleting remaining anti-CD19 CAR T-cells from potentially cured patients and eliminating insertional mutagenesis risk.

### Remission or cure―confirmation of clinical status in larger cohorts

Given there are very few immunotherapies in which the therapy itself is still active and can be readily identified many years later, these patients presented a remarkable opportunity. This raises the prospect of assessing CAR T-cell profiles in larger future clinical cohorts to determine if similar phenotypes and long-term responses hold across other CLL patients, as well as other malignancies. In addition, future development of more advanced ultrasensitive techniques can contribute to better defining the disease status and further determining whether a given patient is actually cured at a given point in time after infusion of CAR T-cells.

## Abbreviations

CAR: chimeric antigen receptor
CLL: chronic lymphocytic leukemia
CR: complete remission
MRD: minimal residual disease
TSCM: T-stem cell memory

## Declarations

### Copyright

© The Author(s) 2023
